# Short-term effects of synchronized vs. non-synchronized NIPPV in preterm infants: study protocol for an unmasked randomized crossover trial

**DOI:** 10.1186/s13063-021-05351-0

**Published:** 2021-06-14

**Authors:** Francesco Cresi, Federica Chiale, Elena Maggiora, Silvia Maria Borgione, Mattia Ferroglio, Federica Runfola, Giulia Maiocco, Chiara Peila, Enrico Bertino, Alessandra Coscia

**Affiliations:** 1 Neonatal Intensive Care Unit, Department of Public Health and Pediatrics, University of Turin, Turin, Italy; 2 Department of Public Health and Pediatric Sciences, Postgraduate School of Pediatrics, University of Turin, Turin, Italy

**Keywords:** Non-invasive ventilation, NIPPV, SNIPPV, Synchronization, Cardiorespiratory events, Preterm infants, RDS

## Abstract

**Background:**

Non-invasive ventilation (NIV) has been recommended as the best respiratory support for preterm infants with respiratory distress syndrome (RDS). However, the best NIV technique to be used as first intention in RDS management has not yet been established.

Nasal intermittent positive pressure ventilation (NIPPV) may be synchronized (SNIPPV) or non-synchronized to the infant’s breathing efforts.

The aim of the study is to evaluate the short-term effects of SNIPPV vs. NIPPV on the cardiorespiratory events, trying to identify the best ventilation modality for preterm infants at their first approach to NIV ventilation support.

**Methods:**

An unmasked randomized crossover study with three treatment phases was designed. All newborn infants < 32 weeks of gestational age with RDS needing NIV ventilation as first intention or after extubation will be consecutively enrolled in the study and randomized to the NIPPV or SNIPPV arm. After stabilization, enrolled patients will be alternatively ventilated with two different techniques for two time frames of 4 h each. NIPPV and SNIPPV will be administered with the same ventilator and the same interface, maintaining continuous assisted ventilation without patient discomfort.

During the whole duration of the study, the patient’s cardiorespiratory data and data from the ventilator will be simultaneously recorded using a polygraph connected to a computer.

The primary outcome is the frequency of episodes of oxygen desaturation. Secondary outcomes are the number of the cardiorespiratory events, FiO_2_ necessity, newborn pain score evaluation, synchronization index, and thoracoabdominal asynchrony. The calculated sample size was of 30 patients.

**Discussion:**

It is known that NIPPV produces a percentage of ineffective acts due to asynchronies between the ventilator and the infant’s breaths. On the other hand, an ineffective synchronization could increase work of breathing. Our hypothesis is that an efficient synchronization could reduce the respiratory work and increase the volume per minute exchanged without interfering with the natural respiratory rhythm of the patient with RDS. The results of this study will allow us to evaluate the effectiveness of the synchronization, demonstrating whether SNIPPV is the most effective non-invasive ventilation mode in preterm infants with RDS at their first approach to NIV ventilation.

**Trial registration:**

ClinicalTrials.gov NCT03289936. Registered on September 21, 2017.

**Supplementary Information:**

The online version contains supplementary material available at 10.1186/s13063-021-05351-0.

## Background

Respiratory problems are some of the major issues to deal with in preterm infants [1].

Because of the immaturity of respiratory mechanisms and structures, the use of support devices is often necessary. These include both conventional mechanical ventilation (MV) techniques, which require the use of an endotracheal tube, as well as non-invasive ventilation (NIV) techniques that use softer ventilator-patient interfaces. Increasing attention is being paid to the latter ones as they are less aggressive and associated with better outcomes both in terms of mortality and short and long-term complications, such as bronchopulmonary dysplasia (BPD) [2, 3].

Nasal intermittent positive pressure ventilation (NIPPV) is a NIV technique in which the infant’s airways are kept open between two pressure levels: peak inspiratory pressure (PIP) and positive end-expiratory pressure (PEEP) [4, 5]. The frequency and duration of each phase are defined by setting the inspiratory and expiratory times or the ventilation rate.

This technique has already shown its superiority in terms of reduced duration of MV, reduced necessity of intubation, decreased extubation failure, and reduced prevalence of BPD if compared with non-invasive techniques based on continuous pressure support, such as continuous positive airway pressure (CPAP) [6]. Recent meta-analyses of studies where NIPPV was used as an alternative to CPAP following extubation show that it reduces the need for re-ventilation and air leaks, without reducing BPD [7]. There is insufficient evidence to recommend NIPPV as the primary mode of respiratory support in the delivery room [1].

It is important to specify that the ventilation rate on NIPPV does not reflect the real infant’s spontaneous respiratory rate (RR), as the ventilator supplies the PIP regardless of respiratory efforts. In order to reproduce a more physiological and gentle ventilation, new devices have been developed able to detect the infant’s respiratory effort and consequently supply a PIP, synchronizing the ventilation rate with the infant’s RR.

The devices used for synchronization can identify the infant’s respiratory effort by detecting variation in flow or pressure. While the MV circuit can detect the exact beginning of inspiration through the continuous monitoring of pressure or through the precise interception of inspiratory and expiratory flow, some difficulties occur in NIV where, as a consequence of the impossibility to detect expiratory flow, the moment of the exact beginning of spontaneous inspiration is hard to identify.

Recently, a new type of NIV ventilator equipped with a pressure sensor has been put on the market. The software of this ventilator is able to calculate the flow according to the pressure variations of the circuit and to capture the flow variations induced by spontaneous breathing, allowing synchronization of the flow with the patient’s respiratory acts [8].

The use of a synchronized NIV technique would allow for a more physiological respiratory support, reducing respiratory fatigue and improving the infant’s compliance. Despite these premises, the diffusion of synchronized NIPPV (SNIPPV) in neonatal intensive care units (NICUs) and studies on its efficacy are limited [9].

Some authors have already demonstrated the benefits of using a synchronized NIV technique in terms of extubation success rate, BPD prevalence, mortality, and neurocognitive development [2, 8]. SNIPPV seems more effective than NIPPV and nasal CPAP (NCPAP) in reducing the need for intubation in respiratory distress syndrome (RDS), in improving the success of extubation, and in treating apnea of prematurity, with a reassuring absence of relevant side effects [7, 8, 10]. SNIPPV delivered through a ventilator can reduce extubation failure but may not confer long-term advantages such as reduction in BPD [1]. Other reported beneficial aspects of SNIPPV include improved thoracoabdominal synchrony, reduced work of breathing (WOB), and reduced need for intubation [5, 7, 11].

It has already been shown that SNIPPV is more effective than NIPPV and NCPAP in reducing the number of desaturations and apneas in preterm infants undergoing CPAP treatment for apnea of prematurity [12]. However, the effectiveness of SNIPPV compared to NIPPV in preterm infants with RDS is still not completely clear.

Our study protocol was designed to evaluate the short-term effects of SNIPPV vs. NIPPV on the major cardiorespiratory variables, trying to identify the best ventilation modality for preterm infants at their first approach to NIV ventilation support, on the bases of cardiorespiratory events reduction and fraction of inspired oxygen (FiO_2_) request.

## Methods

### Aims

Evaluate the short-term effects of SNIPPV vs. NIPPV in a group of preterm infants on the cardiorespiratory events at their first approach to NIV ventilation as first intention (soon after birth) or after extubation.

### Study design and setting

The study has been designed as an unmasked randomized crossover study. It will involve the NICU of the University of Turin. The data analysts will be masked.

### Inclusion criteria

All newborn infants with RDS needing NIV ventilation (NIPPV or SNIPPV) as first intention or after extubation and with all following characteristics will be consecutively enrolled in the study:

1. Gestational age (GA) at birth < 32 weeks
2. First approach to NIV ventilation (first intention or after extubation)
3. Parental written consent
4. ≤ 7 days of life

### Exclusion criteria

The following are the study exclusion criteria:

1. Neurological (including intraventricular hemorrhage (IVH) > 2° grade) or surgical diseases
2. Sepsis (clinical or laboratory-confirmed)
3. Chromosomal or genetic abnormalities
4. Major malformations and congenital anomalies
5. Cardiac problems (including hemodynamically significant patent ductus arteriosus (PDA))
6. Contraindication to NIV (i.e., nasal trauma and gastrointestinal surgery within the previous 7 days).

### Recruitment and randomization

Both parents will sign an informed written consent, and sufficient time will be allowed for consent. Non-Italian-speaking parents will only be asked for their consent if an adult interpreter is available. To support the involvement of participants whose first language is not Italian, a trusted interpreter and a cultural mediator will be used.

The decision to use a NIV support will be based on clinical evaluation. For patients enrolled at NICU admission (first intention subgroup), infants will be enrolled as soon as possible in their first 24 h of life. For patients enrolled after extubation (after extubation subgroup), infants will be enrolled at the moment of extubation. At the start of NIV, eligible patients will be allocated to one of the two arms (NIPPV-SNIPPV-NIPPV or SNIPPV-NIPPV-SNIPPV) by block randomization. Custom software will be used to obtain an arbitrary sequence to randomize patients to both arms, creating balance between patients needing NIV as first intention or after extubation.

After 2 h of stabilization (stabilization phase) in the randomly assigned NIV mode, enrolled patients will be alternatively ventilated with the two different techniques for two time frames of 4 h each. If surfactant is needed before starting the study, the stabilization phase will last 4 h after administration.

Infants will be kept supine throughout the study. During the whole study duration (including the stabilization phase), all patients will be continuously monitored with a multiparametric monitor and the data from the ventilator will also be recorded. The first hour of each NIPPV/SNIPPV time frame will be considered as the wash-out phase and named “adaptation phase”: the data recorded during this phase will be excluded from the analysis. Milk meals will be administered during the adaptation phase.

Pain and compliance scales will be filled in by nurses every 60 min. Blood gas analysis (BGA) values will be recorded at the end of the stabilization phase, at the end of phase A (first NIV modality), and phase B (second NIV modality).

Patients will drop out of the study in case of:

1. NIV failure criteria: FiO_2_ > 40%, pH < 7.2, pCO_2_ > 65 mmHg, ≥ 3 episodes of desaturations (transcutaneous O_2_ saturation (SatO_2_ TC) < 80%) per hour, ≥ 3 episodes of apnea (> 20 s) and/or bradycardia (heart rate (HR) < 80 beats per minute (bpm)) per hour, Silverman score > 6. Necrotizing enterocolitis, bowel perforation, and hemodynamic instability are indications of NIV failure [13]
2. Air leak syndrome (i.e., pneumothorax)
3. Need for invasive procedures during the study
4. Need for surfactant during the study
5. Development of hemodynamic instability or surgical problems during the study
6. Death

Data obtained from dropouts (patients who drop out of the study) will be analyzed separately.

After 8 h of study (and 2 h of stabilization phase), each patient will be ventilated with the best NIV modality according to clinical data and the cardiorespiratory parameters observed during the study.

**Fig. 1:**
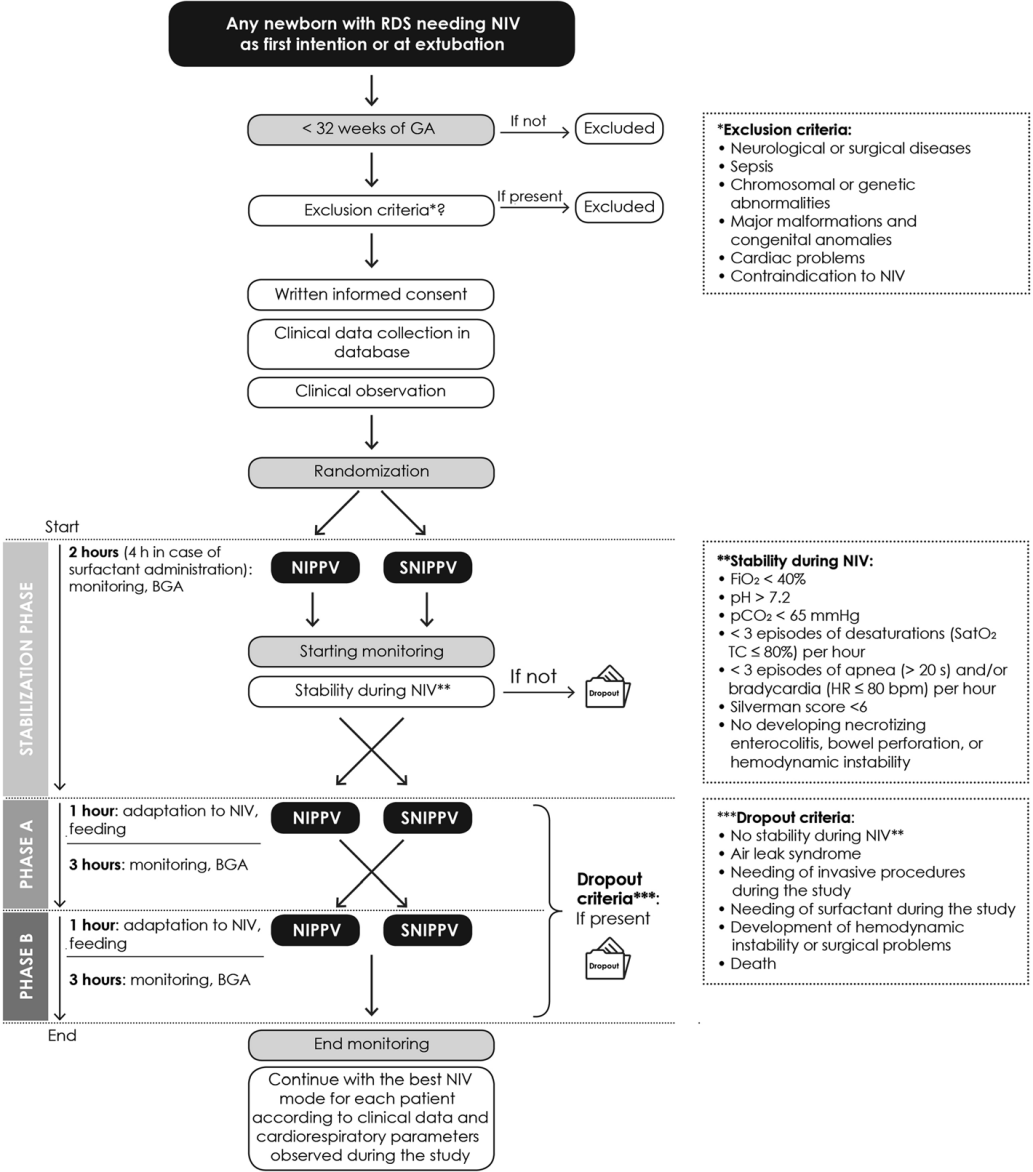
Study design

The design of the study is outlined in Fig. 1.

### Techniques

#### NIV ventilator

NIPPV is a non-invasive ventilation technique in which PIP administration is not synchronized with the infant’s respiratory efforts. SNIPPV is a non-invasive ventilation technique in which PIP administration is synchronized with the infant’s respiratory efforts.

NIPPV and SNIPPV will be delivered using a Giulia® neonatal ventilator via nasal prongs (Ginevri Medical Technologies, Rome, Italy). With this device, clinicians can switch from NIPPV to SNIPPV mode and vice versa without changing the circuit and the ventilation interface avoiding discomfort for the patient. The size of the nasal prongs will be determined by the infant’s weight and characteristics to minimize nasal air leaks as per the manufacturer’s recommendation. In SNIPPV mode, the synchronization will be achieved through an algorithm based on flow detection through a fixed orifice pneumotachograph (2.5 mm inner diameter—dead space 1 mL) interposed between the prongs and the Y piece (flow-SNIPPV).

The optimal NIV setup will be decided by the clinicians and individualized for each patient during the stabilization phase in order to obtain the lowest FiO_2_ levels necessary to reach SatO_2_ TC of 90–94% then the values of PEEP, PIP, backup RR, flow, and inspiratory time will be kept constant during phases A and B.

#### Polygraphy

The Embletta® MPR PG (PG (Multi Parameter Recorder - Polygraphy) - XS is a multiparametric polygraph that records the patient’s ECG trace (to calculate HR), FiO_2_, pulse-oximeter (to obtain HR and SatO_2_ TC), respiratory curve (thoracic impedance, through which RR is calculated). The Embletta® will also be interfaced with Giulia® ventilator using a custom interface (Ginevri Medical Technologies, Rome, Italy) that will obtain three analog output channels (trigger, pressure, flow) from the Giulia® ventilator.

Data from Embletta® will be analyzed by a designed software provided by the manufacturer.

### Monitoring and data collection

Data on respiratory support and overall clinical status will be collected from enrolment to discharge. Data will be recorded continuously by the polygraph from enrolment to the end of the study.

All the data that will be collected will be obtained from the clinical records and from the Embletta® MPR PG-XS. Data will be recorded on a database specifically designed for this study. Access to the database will be password protected. Participants will be identified by trial number only.

Data obtained from the clinical records cannot be modified after being recorded in the database. Data from the Embletta® MPR PG-XS will be recorded and analyzed by software provided by the manufacturer and cannot be modified.

All data recorded throughout the study period are listed in Table 1.

**Table 1:** Data recorded before and during the study period

Before enrolment	During the study period
ANAMNESTIC VARIABLES - GA at birth - Birth weight - Delivery type - APGAR at 1/5 min (and 10 min if available) - Presence of intrauterine growth restriction - Maternal administration of magnesium sulfate - Steroid prenatal prophylaxis (number of doses) - Intrapartum antibiotic prophylaxis (if indicated) - Presence of intraamniotic infection and administration of intrapartum antibiotic therapy CLINICAL VARIABLES - Surfactant administration (time and number of doses) - Type and duration of MV previously administered (if any) - Type and duration of NIV previously administered (if any) - Corrected GA at enrolling - Caffeine doses administered (if any)	CARDIORESPIRATORY VARIABLES - FiO_2_ to maintain SatO_2_TC 90-94% (as weighted mean) - NIV failure^a^ and endotracheal intubation - Number of the cardiorespiratory events^b^ OTHER POLYGRAPH VARIABLES (continuous monitoring) - HR - RR - SatO_2_ TC - thoracic impedance - from ventilator: trigger, pressure, flow - pressure in ventilator circuit CLINICAL VARIABLES - Neonatal Pain Scale score LABORATORY VARIABLES - BGA values at the end of stabilization phase, phase A and phase B

*GA* gestational age, *MV* mechanical ventilation, *NIV* non-invasive ventilation, *SatO*_*2*_ *TC* transcutaneous O_2_ saturation, *HR* heart rate, *RR* respiratory rate

^a^NIV failure criteria: FiO_2_ > 40%, pH < 7.2, pCO_2_ > 65 mmHg, ≥ 3 episodes of desaturations (SatO_2_ TC < 80%) per hour, ≥ 3 episodes of apnea (> 20 s) and/or bradycardia (HR < 80 bpm) per hour, Silverman score > 6. Necrotizing enterocolitis, bowel perforation, and hemodynamic instability are indications of NIV failure

^b^Cardiorespiratory events are defined as episodes of apnea lasting more than 20 s or over 5 s if followed by desaturation or bradycardia and/or episodes of desaturation with blood oxygen saturation below 80% for 4 s or more and/or episodes of bradycardia with HR below 80 bpm.

### Cardiorespiratory events

Cardiorespiratory events are defined as episodes of apnea lasting more than 20 s or over 5 s if followed by desaturation or bradycardia and/or episodes of desaturation in which blood oxygen saturation falls below 80% for 4 s or more and/or episodes of bradycardia with HR below 80 bpm.

### Outcomes

The primary outcome of the study is the frequency of episodes of desaturation.

Secondary outcomes are listed below:

1. The number of the cardiorespiratory events (apnea, bradycardia, oxygen desaturation)
2. FiO_2_ needing during SNIPPV vs. NIPPV monitoring to maintain SatO_2_ TC between 90 and 94%, defined as mean fraction of inspired oxygen, expressed in percentage
3. Newborn pain score evaluation during SNIPPV vs. NIPPV monitoring, using Neonatal Pain Scale Score
4. Synchronization index, defined as the percentage of spontaneous breaths supported by the ventilator
5. Patient-ventilator concordance, defined as the time between the onset of the patient’s inspiratory effort and mechanical inflation in synchronized ventilation
6. Thoracoabdominal asynchrony, defined as the phase difference between thoracic and abdominal impedance

Dropout patients will be considered to establish the frequency of NIV failure, NIV weaning, and the need for surfactant during each phase of the study.

The main result will be the difference in cardiorespiratory events during SNIPPV versus NIPPV.

Tolerance to each of the two NIV modalities will be evaluated by evaluating the number of failure episodes and the cardiorespiratory events and analyzing the scores for individual compliance and pain. The individual need for oxygen under the two NIV modalities will be considered a known risk factor for premature retinopathy and various other complications.

**Fig. 2:**
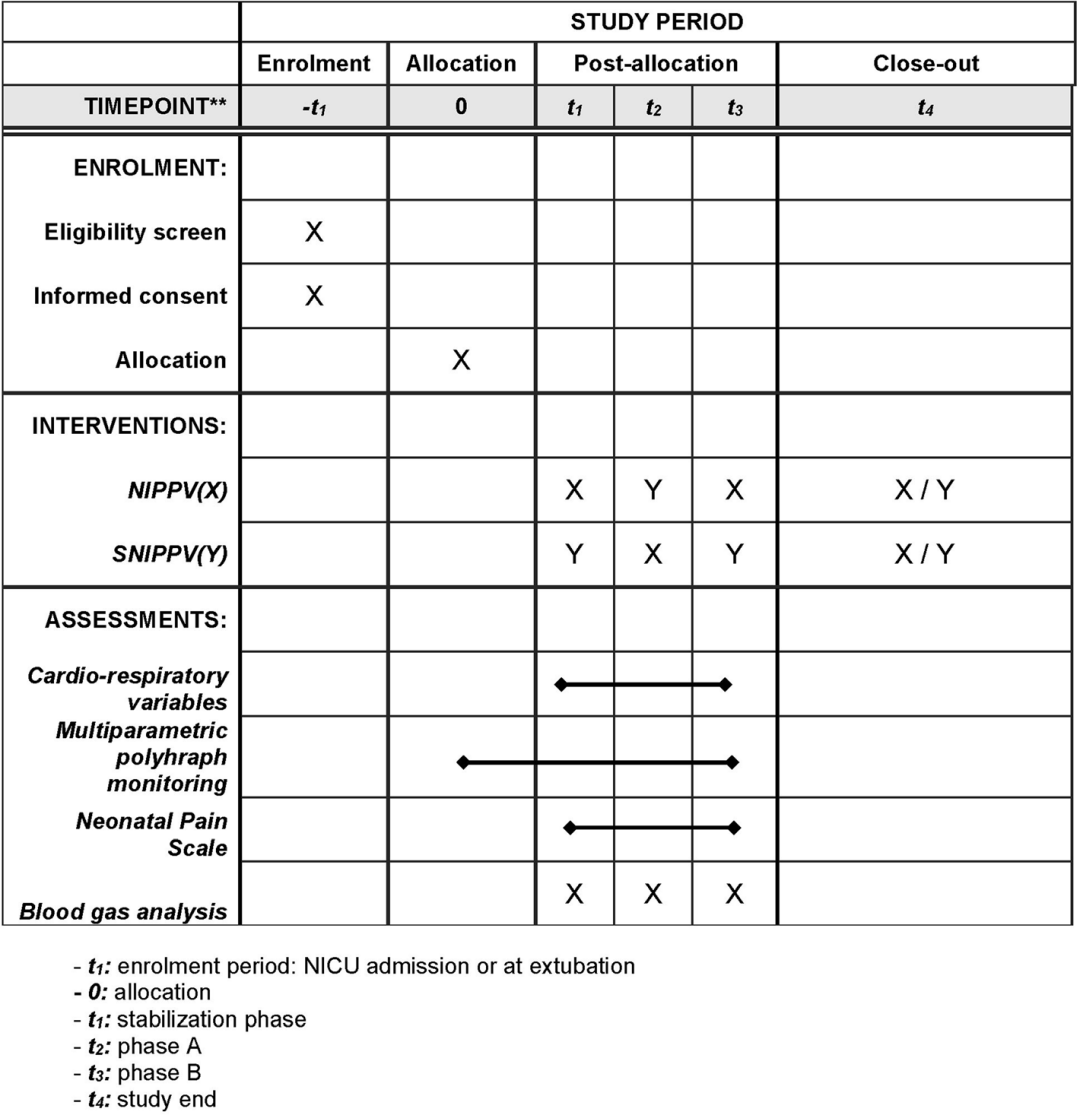
Spirit figure

The Standard Protocol Items: Recommendations for Interventional Trials (SPIRIT) figure of enrolment, interventions, and assessments is shown in Fig. 2. The SPIRIT checklist is provided as Additional file 1.

### Statistical analysis and sample size

Descriptive variables will be analyzed in function of their distribution. T student test or Mann Whitney U test in case of continuous variables (if normally or not normally distributed respectively) and chi-squared or fisher test for qualitative ones. All tests will be two-sided with a significance threshold of 0.05.

A subgroup analysis will be performed according to the time of NIV support (first intention or after extubation).

The number of patients to be enrolled is calculated considering clinically relevant a difference of 30% in cardiorespiratory events between the two ventilation modalities. Assuming a mean of 5 and a SD of 1.5 events/h (based on available literature data), the number of patients to be enrolled is 30, to obtain an 80% power and a significance threshold of 0.05.

## Quality control and quality assurance procedures

### Compliance to protocol

Compliance will be defined as full adherence to protocol. Compliance with the protocol will be ensured by a number of procedures as described.

### Site setup

Principal investigators will participate in preparatory meetings in which details on study protocol, non-invasive ventilation, multiparametric polygraph, and data collection will be accurately discussed with all NICU staff, including nursing and medical staff.

### Safety

Safety endpoints will include incidence, severity, and causality of reported significant adverse events (SAEs). All SAEs will be followed until satisfactory resolution or until the investigator responsible for the care of the participant deems the event to be chronic or the patient to be stable. All expected and unexpected SAEs, whether or not they are attributable to the study intervention, will be reviewed by the principal investigators to determine if there is a reasonable suspected causal relationship with the intervention. If the relationship is reasonable, SAEs will be reported to the ethics committee and inform all other investigators to guarantee the safety of the participants.

## Discussion

The European Consensus Guidelines on the Management of Respiratory Distress Syndrome recommend NIV as the best respiratory support for preterm infants with RDS [1].

It is well known that the NIV is a valid tool to reduce the duration and the need for MV. The reduction in MV is closely related to the development of ventilator lung injury and complications such as BPD. Early switching from the MV to the NIV has been advocated, even for extremely preterm infants [14]. Therefore, it is desirable to identify the best type of NIV to be used at birth or after extubation.

The popularity of NIPPV is rising since its comparison to NCPAP has demonstrated a significant decrease in respiratory failure, re-intubation rates, and extubation failure [6]. However, there is insufficient evidence to recommend NIPPV as the primary mode of respiratory support in the delivery room [1].

Synchronization may be important in delivering effective NIPPV: studies using SNIPPV and delivering NIPPV to infants by a ventilator observed benefits more consistently [7].

The periodic breaths of NIPPV increase tidal volume leading to enhanced removal of CO_2_, sustained alveolar ventilation during episodes of apnea and increased functional residual capacity (FRC) [4, 15]. Asynchronies between ventilator and infant breaths may alter spontaneous breath rhythm, induce glottal narrowing, increase WOB, increase abdominal distension, and cause volutrauma. SNIPPV seems to be associated with an increase in tidal and minute volumes compared to NCPAP [16]. It has also been demonstrated that SNIPPV recruits collapsed alveoli, thereby increasing FRC and decreasing the need for MV [17]. Several explanations may account for the effectiveness of SNIPPV: decreased thoracoabdominal motion asynchrony and flow resistance through the nasal prongs, with improved stability of the chest wall and pulmonary mechanics, increased flow delivery in the upper airway with the addition of a PIP above PEEP [18].

Synchronization during nasal ventilation is considered to provide more efficient respiratory support and synchrony. One of the first studies to demonstrate WOB reduction in preterm infants using SNIPPV was conducted more than ten years ago [19]. Huang et al. [20] supported these benefits of synchronized ventilation achieved by using a Graseby capsule, showing an improved gas exchange and a decreased respiratory effort.

Compared to NCPAP and NIPPV, SNIPPV is more effective in reducing desaturation and apneas; compared to NCPAP, SNIPPV reduces BPD risk, extubation failure, and severe retinopathy of prematurity [16, 21].

Gizzi et al. compared the effects of flow-SNIPPV, NIPPV, and NCPAP on the rates of desaturation and bradycardias in preterm infants with apnoeic-spells (the mean GA at study was 30 weeks) [12]. A randomized crossover study with three treatment phases was conducted: patients received the three modes of ventilation for 4 h each, using a conventional nasal ventilator able to provide synchronization by a pneumotachograph (Giulia® ventilator; GINEVRI srl, Albano Laziale, Rome, Italy). Flow-SNIPPV has been shown to reduce desaturation and apneas in NCPAP infants [12].

SNIPPV may be superior to NIPPV even in patients with RDS, as first intention (soon after birth) or after extubation.

As first intention, flow-SNIPPV combined with surfactant seemed to be a promising strategy for treating infants in the acute phase of RDS [22]. However, a recent meta-analysis showed that early NIPPV appears to be superior to NCPAP alone in decreasing respiratory failure and the need for intubation, without any additional benefits of SNIPPV [10]. Recently, Handoka et al. reported grade of RDS, mean airway pressure, and antenatal steroid use as the predictors of early SNIPPV failure [18].

A lower WOB is an important consideration when choosing which non-invasive mode should be used to support preterm infants immediately after extubation. SNIPPV and NIPPV delivered by a ventilator demonstrated short-term benefits for extubation failure and long-term pulmonary effects for BPD and pulmonary air leaks [7]. SNIPPV post-extubation reduced the WOB and thoracoabdominal asynchrony [5, 11].

Several fans are currently available for SNIPPV. The Giulia® ventilator (GINEVRI srl, Albano Laziale, Rome, Italy) is one of them, providing a flow-SNIPPV. The Giulia® uses a pressure sensor. The trigger quality of this system is not known.

It is known that NIPPV produces a percentage of ineffective acts because they are not synchronized with patient’s acts. On the other hand, a bad synchronization system could have the effect of increasing respiratory work. Our hypothesis is that a valid synchronization could reduce the respiratory work and increase the volume per minute exchanged without interfering with the natural respiratory rhythm of the infant with RDS. A randomized crossover study with three treatment phases will be conducted to evaluate the short-term effects of SNIPPV vs. NIPPV in a group of preterm infants at their first approach to NIV ventilation as first intention or after extubation on the cardiorespiratory events.

The results of this study will allow us to evaluate the effectiveness of the synchronization obtained by the flow-SNIPPV.

The results of this study will demonstrate whether synchronization is the most effective ventilation mode even in preterm infants with RDS.

We want to make a few considerations given the population for this trial is infants with RDS and the current global pandemic of a virus that is especially harmful to pulmonary systems. Since patients of this trial are preterm infants admitted in NICU, existing hygiene rules and standards of care are applied to all patients. Hospital restrictions have a significantly limited parental presence for NICU admitted infants. Otherwise, the trial is not modified. To accommodate risks and restrictions due to COVID-19, all neonates born to mothers with suspected or confirmed COVID-19 who need intensive care are admitted to another unit and are not enrolled in the trial.

## Trial status

The protocol is syncNIPPV17 version no. 1.6. The recruitment is expected to begin on September 1st, 2020. The anticipated date of recruitment completion is August 31st 2021.

The trial was registered in ClinicalTrials.gov on 09/21/2017, NCT03289936.

All items from the WHO Trial Registration Data set can be found within the manuscript.

## Supplementary Information

**Additional file 1.** SPIRIT 2013 checklist: recommended items to address in a clinical trial protocol and related documents.

## Data Availability

The datasets generated during the current study are available from the corresponding author on reasonable request.

## Abbreviations

BGA: Blood gas analysis
BPD: Bronchopulmonary dysplasia
Bpm: Beats per minute
CPAP: Continuous positive airway pressure
flow-SNIPPV: Flow-synchronized nasal intermittent positive pressure ventilation
FiO_2_: Fraction of inspired oxygen
FRC: Functional residual capacity
GA: Gestational age
HR: Heart rate
IVH: Intraventricular hemorrhage
MV: Mechanical ventilation
NCPAP: Nasal continuous positive airway pressure
NICU: Neonatal intensive care unit
NIPPV: Nasal intermittent positive pressure ventilation
NIV: Non-invasive ventilation
PDA: Patent ductus arteriosus
PEEP: Positive end-expiratory pressure
PIP: Peak inspiratory pressure
RDS: Respiratory distress syndrome
RR: Respiratory rate
SatO_2_ TC: Transcutaneous O_2_ saturation
SNIPPV: Synchronized nasal intermittent positive pressure ventilation
WOB: Work of breathing
SAEs: Significant adverse events
